# Supplementary figures and images for: Optimization of Dynamic SSVEP Paradigms for Practical Application: Low-Fatigue Design with Coordinated Trajectory and Speed Modulation and Gaming Validation
Source: Sensors (Basel). 2025 Jul 31;25(15):4727. doi: 10.3390/s25154727 (PMC12349208; doi:10.3390/s25154727)

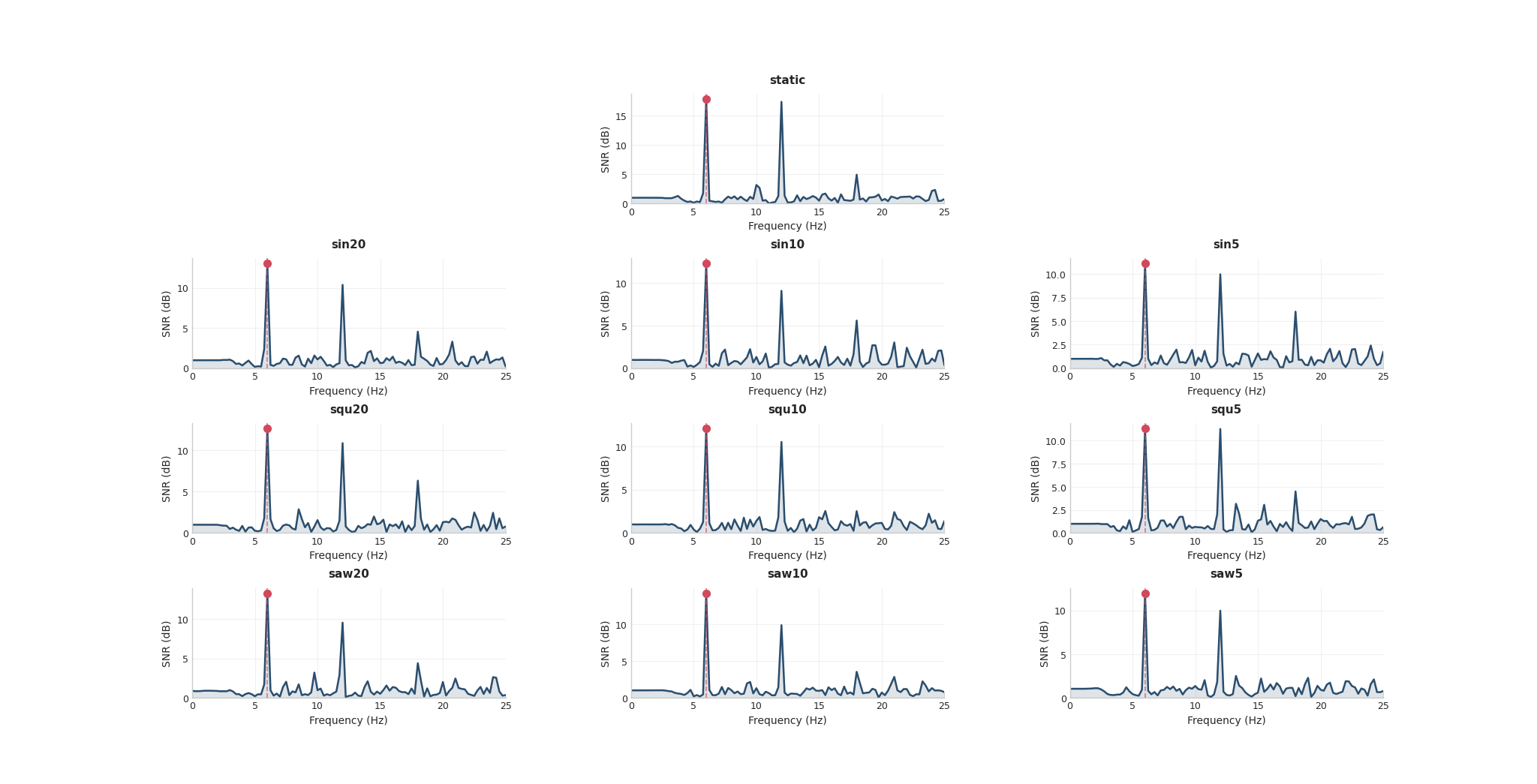

Supplement: Supplementary file 1 [file sensors-25-04727-s001.zip › Supplementary Figure S1.png]

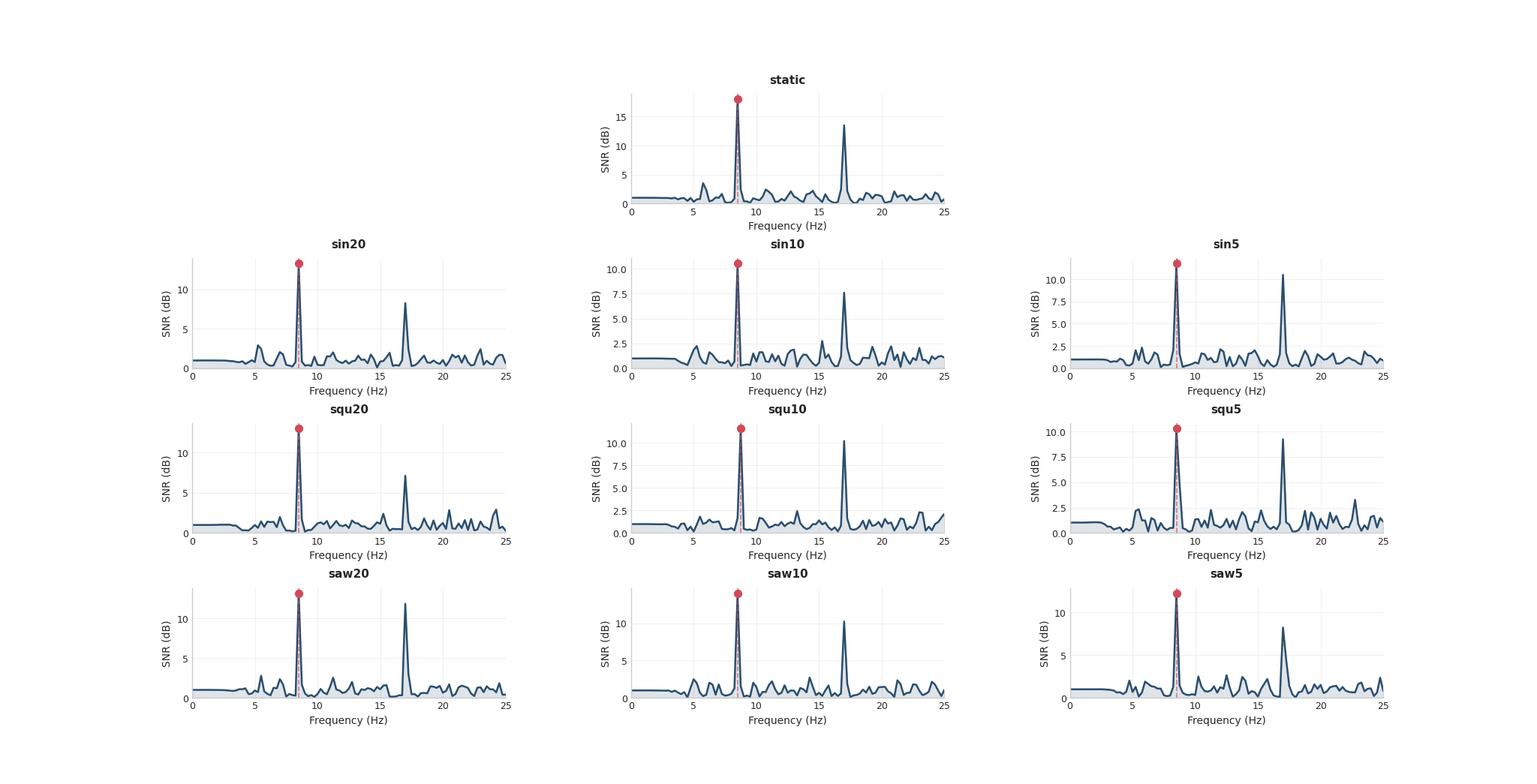

Supplement: Supplementary file 1 [file sensors-25-04727-s001.zip › Supplementary Figure S2.png]

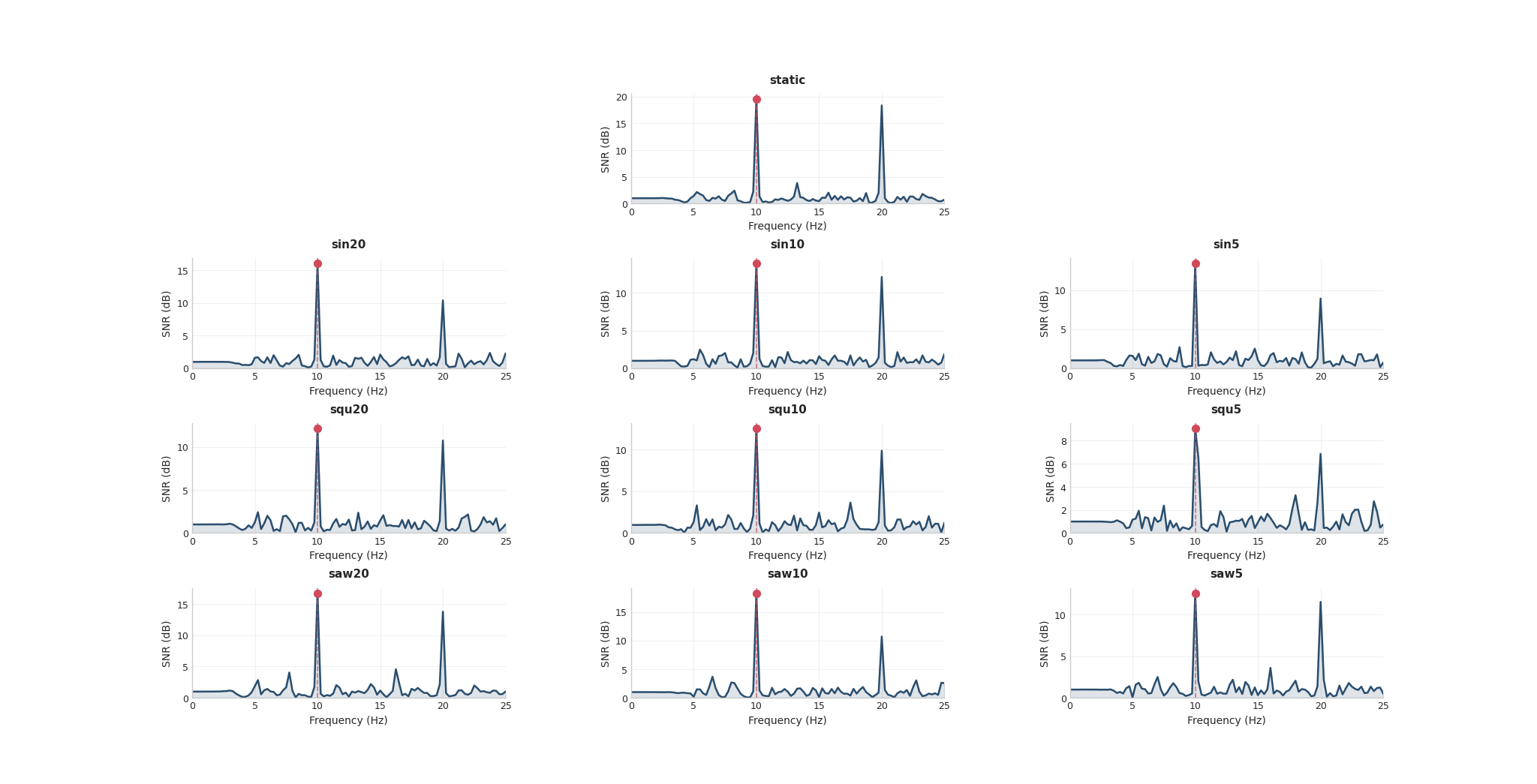

Supplement: Supplementary file 1 [file sensors-25-04727-s001.zip › Supplementary Figure S3.png]

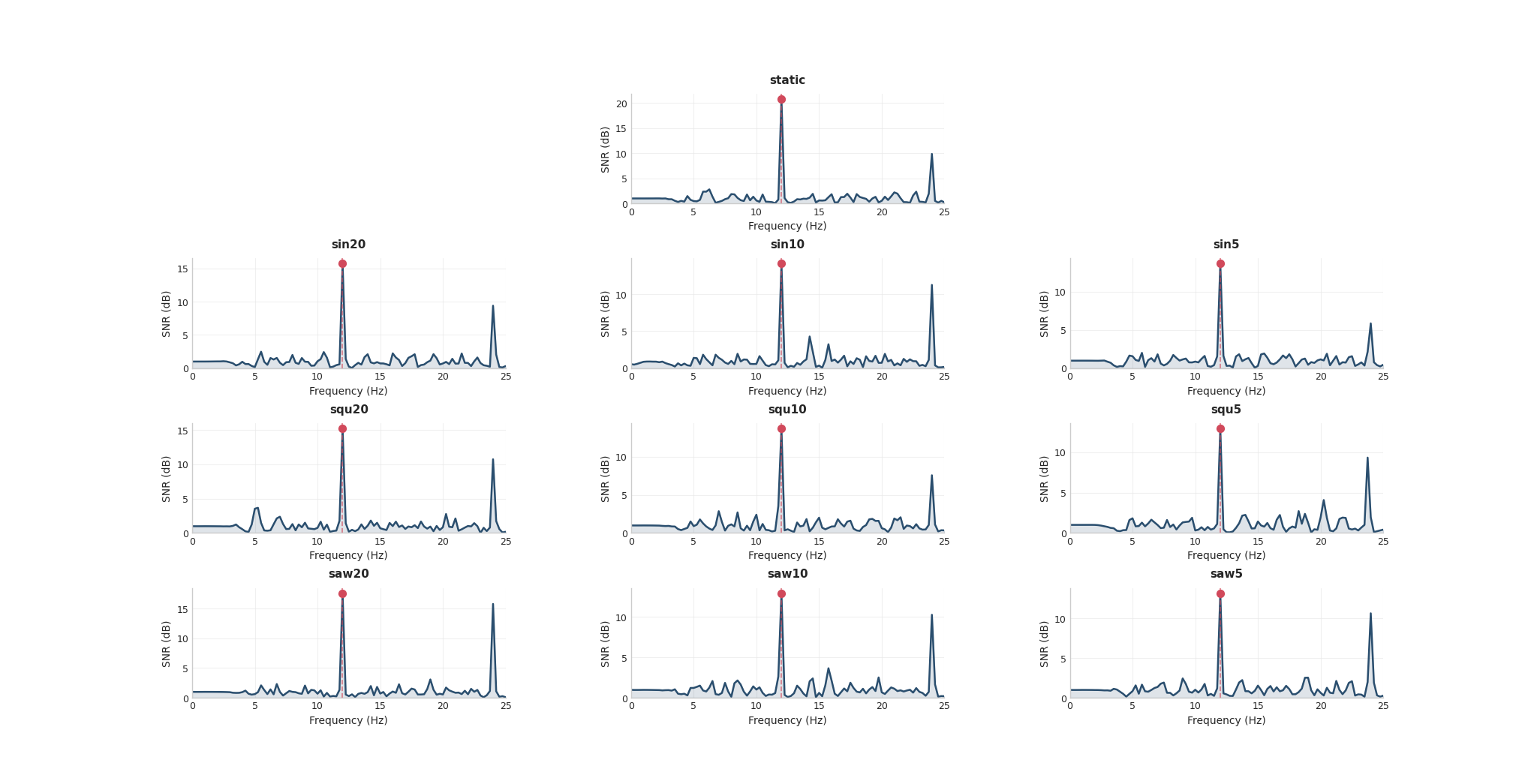

Supplement: Supplementary file 1 [file sensors-25-04727-s001.zip › Supplementary Figure S4.png]
